# Feasibility of endometrial sampling by vaginal tampons in women with Lynch syndrome

**DOI:** 10.1186/s12905-020-00920-y

**Published:** 2020-03-17

**Authors:** Jorien M. Woolderink, Geertruida H. De Bock, Bettien M. van Hemel, Erwin Geuken, Harry Hollema, Naomi Werner, Marian J. Mourits

**Affiliations:** 1 Department of Gynecology, Martini Hospital, Groningen, the Netherlands; 2 Department of Gynecologic Oncology, University of Groningen, University Medical Center Groningen, P.O. Box 30.001, 9700 RB Groningen, the Netherlands; 3 Department of Epidemiology, University of Groningen, University Medical Center Groningen, Groningen, the Netherlands; 4 Department of Pathology, University of Groningen, University Medical Center Groningen, Groningen, the Netherlands; 5 Department of Pathology, Martini Hospital, Groningen, the Netherlands

**Keywords:** Endometrial sampling, Tampons, Lynch syndrome, Surveillance, Endometrial cancer

## Abstract

**Background:**

Endometrial sampling for the surveillance of women with Lynch syndrome is an invasive and painful procedure. The aim of this study was to evaluate the feasibility of a less invasive procedure of collecting vital cells by vaginal tampons.

**Methods:**

This was a prospective feasibility study of women scheduled to undergo annual gynecological surveillance, including endometrial sampling. We included consecutive asymptomatic women with Lynch syndrome or first-degree relatives and asked them to insert a vaginal tampon 2–4 h before attending their outpatient appointment. Feasibility was evaluated by the following metrics: patient acceptance, pain intensity of each procedure (assessed by visual analog scale; range 0–10), and the presence of vital cells obtained by tampon-based or endometrial sampling methods. Two pathologists independently evaluated all samples.

**Results:**

In total, 25 of 32 approached women completed the tampon-based procedure, with 23 of these subsequently undergoing invasive endometrial sampling. The median visual analog scale scores for tampon use and invasive endometrial sampling were 0 (range, 0–10) and 5.5 (range, 1–10) (*p* < 0.001). None of the tampon samples analyzed by cytology showed endometrial cells, but they did contain vital squamous cells and granulocytes. By contrast, 18 (78%) of the invasive endometrial samples contained enough endometrial tissue for analysis. No endometrial abnormalities were found by endometrial sampling.

**Conclusions:**

Tampon-based endometrial surveillance was a well-accepted and non-painful procedure, and although tampons contained vital cells, they did not provide endometrial cells. However, this study was limited to asymptomatic women with Lynch syndrome (no endometrial pathology), indicating that research is needed to evaluate whether the tampon method has any utility for endometrial surveillance in women with Lynch syndrome.

## Background

Lynch syndrome (LS) carries a lifetime risk of 10–55% for endometrial cancer depending on the gene mutation carried [1–7]. The mean age for developing cancer in LS is 50–55 years, which is 10 years younger than in sporadic cases [3–5, 8]. Women with LS may therefore benefit from annual surveillance to detect endometrial abnormalities at premalignant or early malignant stages [8–12]. However, this has not been shown to improve survival [13].

There is ongoing debate as to whether endometrial surveillance should be performed by transvaginal ultrasound (TVU) with standard endometrial sampling or by TVU alone, reserving endometrial sampling for when it is clearly indicated [8, 10–13]. Signs of abnormal perimenopausal bleeding can be misinterpreted and endometrial cancer can be missed [14]. The current Dutch guideline for LS advises annual endometrial surveillance by TVU plus standard endometrial sampling between the ages of 40 and 60 years. A major disadvantage of such sampling is that it is invasive and painful, irrespective of the indication [15, 16]. An alternative strategy to avoid this pain is to perform hysteroscopy, typically with colonoscopy, while under conscious sedation [15, 17, 18]. Given the pain and invasiveness of these procedures, we wanted to identify an alternative, less painful, and non-invasive method for collecting endometrial cells. One option may be to use vaginal tampons.

Obtaining cells from vaginal tampons for diagnosis has been reported previously for women with cervical cancer, high-grade serous ovarian cancer, and endometrial cancer [19–25]. In 1954, it was first described that malignant cells could be obtained from tampons used in women with cervical and endometrial cancer [19, 20]. Much later, in 2004, a study was performed that analyzed methylated DNA in cervical-vaginal secretions from women with and without endometrial cancer obtained by tampons [23]. Compared to women without endometrial cancer (who underwent a hysterectomy for benign reasons), those with endometrial cancer presented higher levels of methylated genes in the vaginal secretions obtained by tampons [23]. Recently, in 2015, Bakkum-Gamez et al. reported a study of tampon-based screening for 66 women before hysterectomy for endometrial cancer (*n* = 38) or benign reasons (*n* = 28). Gene level analysis of the tampon samples showed a significantly higher methylation rate in women with endometrial cancer compared to women with benign endometrial abnormalities [25]. None of these existing studies has reported on the tolerability of tampon use for cell collection [19–25]. Moreover, studies to date have only reported the use of tampons in women with ovarian or cervical malignancies and none has considered its use for endometrial surveillance in asymptomatic women with LS.

In this study, we assessed the feasibility of collecting endometrial cells with tampons during annual surveillance in women with LS. The primary aim was to assess whether tampon use is less painful than the current invasive procedure of endometrial sampling. The secondary aim was to evaluate whether it is possible to identify endometrial cells from tampons in asymptomatic women with LS, and if so, whether these can be analyzed by a pathologist.

## Methods

### Study design

This was a prospective pilot study of consecutive women who underwent annual gynecological surveillance for LS at either a family cancer clinic (University Medical Center Groningen) or a gynecology outpatient clinic (Martini Hospital), Groningen, from January 2017 until August 2017. Written informed consent was obtained from all participants. The ethics committee of the University Medical Center in Groningen approved the study. All relevant data were entered into a separate password-protected database, and patient identities were protected by assigning study-specific unique patient numbers.

### Inclusion criteria

We included all asymptomatic pre- and postmenopausal women with LS women (i.e., proven carriers of a pathogenic mutation in either *MLH1, MSH2, MSH6*, or *PMS2*). We also included first-degree relatives at a 50% risk of the gene mutation. Participants were required to have no known endometrial abnormalities at the time of annual gynecological surveillance.

### Collection of endometrial cells by tampon or endometrial sampling

All women who met the inclusion criteria received written information about the study. If they agreed to participate, as indicated by the return of a signed informed consent form, we sent a standard size cotton vaginal tampon with a 10 mL bottle of saline (0.9% NaCl) by post. Women were asked to insert the tampon vaginally 2–4 h before their surveillance visit. This time frame has been reported as optimal for obtaining vital cells of sufficient quality for analysis [22, 25]. Women were instructed to wet the tampon with the 10 mL of saline before insertion. This was to improve cell adherence to the outside of the tampon and to prevent cell dehydration. They were asked not to take a bath or shower after inserting the tampon.

At the outpatient clinic, the tampon was removed by the patient and handed to the gynecologist before starting the physical examination. The gynecologist then immersed the tampon at least ten times in a cytological fixation fluid and sprayed a ThinPrep® cytology preservation solution (Hologic, Cytyc Corp., Marlborough, MA, USA) along the tampon to collect as many cells as possible. After removing the tampon, women were asked to report the pain score for the tampon procedure by visual analog scale (VAS) score, for which zero indicated no pain and ten indicated the most severe pain imaginable. All women were then offered standard gynecological surveillance consisting of a TVU and subsequent endometrial sampling, for which the VAS score was repeated.

### Cytological and histological analysis

The tampon fluid was sent for cytological analysis and the endometrial sample was sent for independent histological analysis at a pathology laboratory, without mention of sample inclusion in the tampon study.

One cytological slide was made for each sample of tampon fluid in a ThinPrep® T5000 Processor (Hologic) and Pap stained. All cytological samples obtained with the tampons were analyzed by an experienced cyto-technician followed by two cyto-pathologists, and the findings were described in reports that were compared. The presence and quality of endometrial cells and the presence or absence of atypia or endometrial cancer were analyzed in the samples.

Endometrial samples were collected with a Pipelle® for histological analysis, before hematoxylin and eosin staining and slide creation. All histopathologic analysis was done according to standard procedures. The presence or absence of hyperplasia, with or without atypia or endometrial cancer, was analyzed by two pathologists and the findings were described in reports that were compared. All samples were stored in the pathology department.

### Data collection

We recorded details of patient characteristics and clinical data for each woman. The clinical data of interest were parity, age at the surveillance visit, type of gene mutation, menopause status, use and type of contraceptive, date of last menstrual period, endometrial thickness at TVU, cytopathology reports of tampon samples, and histopathology reports of endometrial samples. The VAS scores (range, 0–10) for the tampon procedure and for standard endometrial sampling were also recorded.

### Data analysis

Differences in pain by VAS score between the tampon-based and endometrial sampling methods were evaluated by Wilcoxon signed-rank tests. Endometrial cell yield for both procedures was also assessed. All data analysis was performed using IBM SPSS, Version 20 (IBM Corp., Armonk, NY, USA).

## Results

### Participant characteristics

In total, 32 women received information about the tampon study. Of these, 25 (78%) accepted and were included in the analysis, while the remaining 7 (22%) declined to participate (5 premenopausal and 2 postmenopausal). The characteristics of the 25 participants are summarized in Table 1. The median age was 47.0 years (range, 37–71 years), 15 (60%) were premenopausal and 10 (40%) were postmenopausal. In the premenopausal group, three used oral contraceptives, four used a Mirena IUD®, and one used Depo-Provera. Only one woman in the postmenopausal group used hormone replacement therapy. The mean endometrial thicknesses by TVU in the premenopausal and postmenopausal groups were 4.5 mm (range, 2–18 mm) and 3.0 mm (range, 1–7 mm), respectively.

**Table 1:** Characteristics of women with LS during endometrial surveillance by tampons and subsequent endometrial sampling (*n* = 25)

	Overall (*n* = 25)	Premenopausal women (*n* = 15)	Postmenopausal women (*n* = 10)
**Age at study entry, mean (SD) in years**	48.8 (9.9)	42.8 (4.3)	57.9 (9.0)
**Age at study entry, median (range) in years**	47.0 (73–71)	42.0 (37–50)	56.0 (46–71)
**Gene mutation**			
*MLH1*	3 (12%)	2 (13%)	1 (10%)
*MSH2*	5 (20%)	3 (20%)	2 (20%)
*MSH6*	6 (24%)	5 (33%)	1 (10%)
*PMS2*	5 (20%)	2 (13%)	3 (30%)
Lynch	1 (4%)	–	1 (10%)
First degree relative LS	5 (20%)	3 (20%)	2 (20%)
**Menopausal state**			
Premenopausal	15 (60%)	15 (100%)	Na^a^
Postmenopausal	10 (40%)	Na^a^	10 (100%)
**Parity**			
Nulliparous	4 (20%)	2 (13%)	2 (40%)
Primi/multiparous	16 (80%)	13 (87%)	3 (60%)
Unknown	5		5
**Hormonal treatment**			
OC	3 (12%)	3 (20%)	–
Mirena IUD	5 (20%)	4 (27%)	1 (10%)
Progesterone	1 (4%)	1 (7%)	–
HRT^b^	1 (4%)	–	1 (10%)
None	15 (60%)	7 (47%)	8 (80%)
**Endometrial response (mm),** Median (range)	3.8 (1–18)	4.5 (2–18)	3.0 (1–7)
1–2	3 (13%)	1 (6%)	2 (22%)
> 2–4	9 (38%)	6 (40%)	3 (33%)
> 4–6	5 (21%)	2 (13%)	3 (33%)
> 6–8	1 (4%)	–	1 (11%)
> 8–10	2 (8%)	2 (13%)	–
> 10	4 (17%)	4 (27%)	–
Unknown	1	–	1
**Last period**			
- < 2 weeks	3 (12%)	3 (21%)	–
- ≥ 2 weeks	11 (46%)	11 (79%)	–
Postmenopausal	10 (42%)	–	10 (100%)
Unknown	1	1	–

^a^*Na* not applicable

^b^*HRT* hormone replacement therapy

### Pain scores for the tampon and endometrial sampling methods

In total, 25 women underwent tampon-based sampling and 23 of these progressed to invasive endometrial sampling. The VAS scores for each procedure are summarized in Table 2. The median overall VAS scores were 0 (range, 0–10) for the tampon method and 5.5 (range, 0–10) for the endometrial sampling method, and the difference in the level of pain between methods was statistically significant (*p* < 0.001). We also compared VAS scores by menopausal status. Among premenopausal women, the median VAS score was 0 (range, 0–1) for the tampon method and 5.0 (range, 3–9) for the endometrial sampling method; the corresponding median VAS scores in postmenopausal women were 4.5 (range, 0–10) and 5.7 (range, 1–10), respectively.

**Table 2:** Yield of endometrial cells obtained with tampons and standard endometrial sampling

	Tampons (***n*** = 25) (%)	Endometrial sampling (***n*** = 25) (%)
**Median VAS score (range)**	0.0 (0–10)	5.5 (1–10)
Premenopausal women	0.0 (0–2)	5.0 (3–9)
Postmenopausal women	4.5 (0–10)	7.0 (1–10)
**Quality of cells in the samples**		
- Good	25 (100%)	18 (78%)
- No/To less endometrial cells	25 (100%)	5 (22%)
- Endometrial sampling not obtained cervical stenosis fear for pain		1 1
**Sufficient endometrial samples**	0	18 (78%)
Premenopausal women	–	13 (72%)
Postmenopausal women	–	5 (28%)
**Insufficient endometrial samples**	25 (100%)	5 (23%)
Premenopausal women	15 (60%)	1 (20%)
Postmenopausal women	10 (40%)	4 (80%)

### Cytological and histological analysis

No differences were found in the pathological outcomes of either the tampon fluid or the endometrial samplings when comparing the reports of the two pathologists. The results were again compared by the menopausal statuses of women and are summarized in Table 2. Of note, none of the cytological samples obtained with tampons contained endometrial cells or endocervical cylindric epithelial cells, although all samples contained vital granulocytes and squamous cells.

In the premenopausal group, only 13 women (87%) provided endometrial samples with enough endometrial tissue for histopathological analysis, and these contained no endometrial abnormalities. One of the endometrial samples contained insufficient tissue for analysis and one woman refused to undergo the procedure because of severe pain when it was last performed. In the postmenopausal group, five endometrial samples (50%) contained enough endometrial tissue for histopathological analysis, but again, none of these revealed endometrial abnormalities. One woman could not undergo endometrial sampling because of cervical stenosis. In the other four cases, samples produced insufficient yields. Three of these cases had an endometrial thickness of 2–3 mm and one had an endometrial thickness of 7 mm, but no further endometrial tissue analysis was performed in this final case because the woman was asymptomatic.

## Discussion

In this feasibility study of an unselected case series of 25 asymptomatic women who underwent annual gynecological surveillance for LS, tampon use was well accepted and less painful than invasive endometrial sampling. The median VAS score with the tampon method was 0 compared with 5.5 for standard endometrial sampling, and this difference was statistically significant. However, although all tampon-based samples contained vital cells, no endometrial cells were collected. This may relate to the fact that women were asymptomatic and free from endometrial pathology.

Standard endometrial sampling is a painful procedure. In other research of women with LS and of first-degree relatives who underwent annual surveillance, the VAS score for the pain associated with standard endometrial sampling was 5.0, which is comparable to our result of 5.5 [16]. In the same cohort, 20% of women also decided to refrain from endometrial sampling because of pain, opting instead for either preventive surgery or annual gynecological surveillance by TVU alone [16]. Two other studies evaluated the utility of combining endometrial sampling and colonoscopy in women with LS to allow the procedure to be performed under conscious sedation. They concluded that this combination resulted in a less painful experience than endometrial sampling without sedation in a standard office setting [17, 18]. Overall, 78% of the standard endometrial samplings in the present study contained enough tissue for analysis, which is consistent with the levels of 74–90% reported in other studies [26, 27].

All tampon samples in this study contained vital cells. However, we detected no endometrial cells, which contrasts starkly with earlier research [19–25]. The most plausible explanation for this is that we tested endometrial cells in healthy women rather than in women with gynecological (pre)malignancies. Indeed, given that we found no malignancies or endometrial abnormalities, it is plausible that endometrial cells would not be shed.

To date, studies reporting on the collection of endometrial cells with tampons have compared the yields in women with and without endometrial cancer. In those cases, it is found that malignant cells and methylated DNA were present on vaginal tampons obtained from women with endometrial cancer [20–23, 25, 28–35]. DNA methylation in cells is a sensitive procedure for discriminating between women with and without endometrial cancer [23, 25]. Given that DNA methylation assays can be performed on cell fragments and do not require intact cells, they may potentially be more sensitive than the tampon technique used in this study [25].

Other studies found that endometrial cells detected by cervical smear can be used to indicate endometrial pathology, especially when vaginal bleeding is present [36, 37]. However, none of the women in this study had vaginal blood loss during the procedures. Using vaginal tampons to collect cells is a well-accepted procedure, and although three women in the postmenopausal group reported high VAS scores for the procedure (range, 7–9), most reported low scores (range, 0–2), and none of the women in the premenopausal group reported pain. Further research is therefore warranted to determine the applicability of vaginal tampons for surveillance in women at increased risk of endometrial cancer. Women with LS have an increased lifetime risk of developing endometrial cancer, translating to an annual risk of about 2.5%. Given that standard endometrial sampling is invasive and painful, with some opting not to undergo surveillance, there is certainly merit in investigating whether it could be replaced by the tampon method. We now require a larger and longer-term study to clarify the true utility of tampon-based screening in this cohort [1, 3, 5–7, 38–40].

To the best of our knowledge, no prior study has evaluated the feasibility of collecting endometrial cells with tampons in asymptomatic women. Moreover, we are aware of no research assessing this procedure in women with LS. A limitation of the study is that we did not consider whether endometrial abnormalities are identifiable by the tampon method in symptomatic women with LS. Another limitation is that DNA methylation in the cells of the 25 asymptomatic women in this pilot study was not evaluated. Future research should focus on the opportunity to find abnormal endometrial cells by using tampons in women with endometrial (pre)malignancies.

In conclusion, the tampon procedure is a non-painful and well-accepted procedure compared with invasive endometrial sampling during annual gynecological screening for LS. However, endometrial cells were only obtained from asymptomatic women with LS. Further research should now focus on the effectiveness and applicability of vaginal tampons for identifying endometrial abnormalities in the annual screening of women with LS. We advocate that future research should also include DNA methylation assays as part of the sample analysis. If tampon use can be shown to be useful for screening or diagnosis, it could help to avoid painful and invasive procedures, thereby improving uptake.

## Data Availability

All data generated or analyzed during this study are included in this published article [and its supplementary information files].

## Abbreviations

LS: Lynch syndrome
TVU: transvaginal ultrasound
VAS: Visual Analog Scale
